# Covalent Modification by Glyoxals Converts Cytochrome c Into its Apoptotically Competent State

**DOI:** 10.1038/s41598-019-41282-2

**Published:** 2019-03-18

**Authors:** Gurumayum Suraj Sharma, Marina Warepam, Reshmee Bhattacharya, Laishram Rajendrakumar Singh

**Affiliations:** 1 Dr. B. R. Ambedkar Center for Biomedical Research, University of Delhi, Delhi, 110007 India; 2 Department of Botany, Zakir Husain Delhi College, University of Delhi, New Delhi, 110002 India; 3 Department of Biotechnology, Manipur University, Canchipur, Imphal East, Manipur 795003 India

## Abstract

The effects of glycation by glyoxal (Gly) and methylglyoxal (MGly) on the early and late conformational alterations in Cytochrome c (Cyt c) were studied. Spectroscopic measurements revealed that Cyt c undergo certain conformational alterations and exposure of heme upon overnight incubation with Gly and MGly. These were followed by the reduction of heme centre and activation of its peroxidase-like, which is crucial for initiation of the intrinsic apoptotic pathway. An extended incubation resulted in appearance of AGE-like fluorescence, with significant alterations in secondary structural compositions. However, no amyloidogenic conversions were observed as suggested by TEM analyses. The study provides an insight to the role of glycating agents, elevated under oxidative stress in inducing Cyt c release and apoptosis.

## Introduction

Hyperglycemia represents a common hallmark of diabetic complications and is characterized by increased levels of sugars and their metabolites^1^. Gly and MGly are formed via oxidation of reducing sugars under chronic hyperglycemic conditions and endogenous MGly can also be formed from triose phosphate intermediates of glycolysis^2^. These sugars metabolites have high tendency of covalently modifying proteins, via a process termed as “protein glycation”. Such modifications are known to induce protein structural alterations resulting in functional loss and even lead to aggregate/amyloids formation, and have been associated with several age related disorders and neurodegenerative diseases (NDs). Accumulation of advanced glycation end products (AGEs) in plasma as a consequence of glycation is also a primary factor for coronary heart diseases^3–7^.

Most studies on protein glycation have focused largely on identifying toxic oligomers and formation of AGEs^8–12^. However, early structural alterations accompanying such modifications have not yet been thoroughly studied. Cyt c is a multi-functional protein present in inner mitochondrial membrane (IMM). In respiration, it is involved in electron transport (ET), whereas in apoptosis, Cyt c release from IMM is known to activate caspase 9 enzyme, initiating a cascade of reactions. Structural alterations in the protein and disruption of its redox state are known to trigger its pro-apoptotic activity^13–17^ and these changes represent early events in apoptosis^16^. Hence, maintaining its native structure and redox status is crucial for proper cellular function. Considering the fact that alteration in conformation of Cyt c is known to be an early event in driving cells towards apoptosis, it is worthwhile to study the effects of such agents on the early structural transitions of Cyt c. We have investigated the early and late conformation alterations in Cyt c upon glycation by Gly and MGly. Our results suggest that overnight incubation with Gly and MGly resulted in certain tertiary structural changes with exposed heme, disruption of the redox state and heme-Met80 coordination, ultimately resulting in activation of peroxidase function. Upon prolonged incubation, there was significant loss in secondary structure with appearance of AGEs specific fluorescence. Our study provides an insight to the molecular mechanism for the role of glycated Cyt c in eliciting its toxicity.

## Results

### Determination of Cyt c Glycation

**Figure 1:**
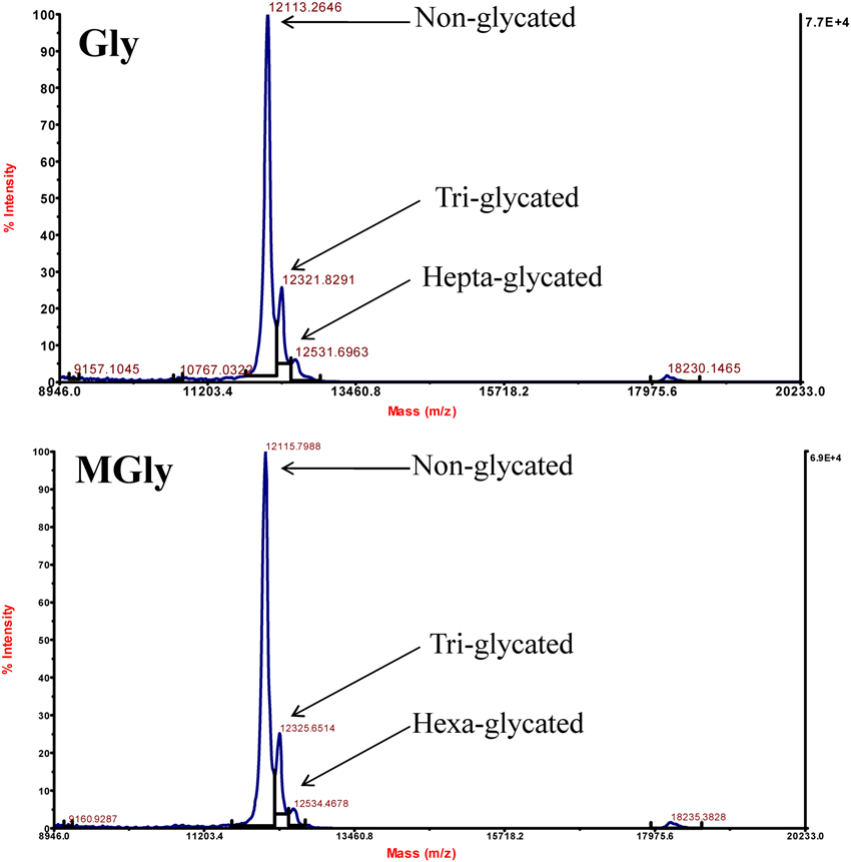
Intact Mass spectrometric analysis of Cyt c treated with 10 mM Gly and MGly.

To determine the extent of glycation, we have carried out carbonyl estimation assay for the untreated and treated Cyt c samples. It is seen in Table 1 that the proteins have been incorporated with Gly and MGly as suggested by increase in carbonyl contents. After overnight incubation, there were 2–4 and 3–5 fold increase for Gly- and MGly-modified Cyt c, respectively. These were further confirmed using MALDI-TOF mass spectrometric analysis (See Fig. 1). For each molecule of Gly or MGly reacting with side chain of Cyt c, there will be an increase of 58 and 74 Da in the protein mass. It is seen in Fig. 1, that incubation of Cyt c with 10 mM Gly/MGly resulted in appearance of two more peaks, in addition to the major peak which corresponds to the non-glycated form of Cyt c. Our result suggests that the protein was glycated at multiple sites, and presents a mixture of non-glycated Cyt c and Cyt c with Gly/MGly linked to 3–7 sites.

**Table 1:** Protein carbonyl content of Cyt c incubated overnight with different concentrations of Gly and MGly.

Concentrations (mM)	Carbonyl content (µM mg^−1^)	
	Gly	MGly
0	65.6	69. 2
0.5	114.4	190.8
1	122.0	200.4
5	282.0	275.2
10	279.6	318.0

### Glycation disrupts Cyt c structure

**Figure 2:**
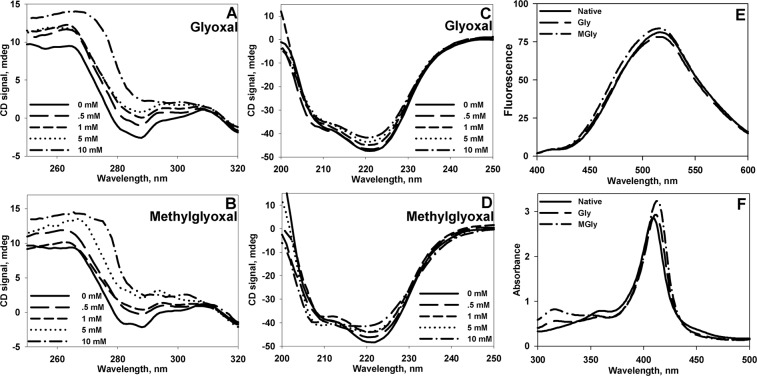
Conformational status of native and glycated Cyt c. Near- (**A**, **B**) and Far- (**B**, **C**) UV CD spectra of Cyt c modified with varying concentration of glyoxals (0–10 mM). Panel E and F show ANS fluorescence and heme absorption spectra of native and glycated Cyt c. To maintain clarity, only the spectra of unmodified and Cyt c modified with 10 mM glyoxals are shown in Panel E,F.

We further assessed the effects of Gly and MGly on early and late conformational alterations of Cyt c. Early conformational changes were followed after overnight incubation (12–14 Hrs) of Cyt c with Gly and MGly. As seen in Fig. 2A,B, glycation results in disruption of native tertiary interactions. In both cases, there is a significant loss in tertiary contacts as suggested by near-UV CD spectra. There were subtle alterations in secondary structural components (see Fig. 2C, D) indicating that the resulting conformation is not a denatured state, but a non-native state with disordered tertiary interactions. However, we did not observe any ANS binding to these non-native structures (Fig. 2E). Visible absorption spectroscopic measurement further revealed that there is a slight increase in the absorption of heme (409 nm) indicating partial heme exposure to solvent (Fig. 2F).

### Redox status of Cyt c upon glycation

**Figure 3:**
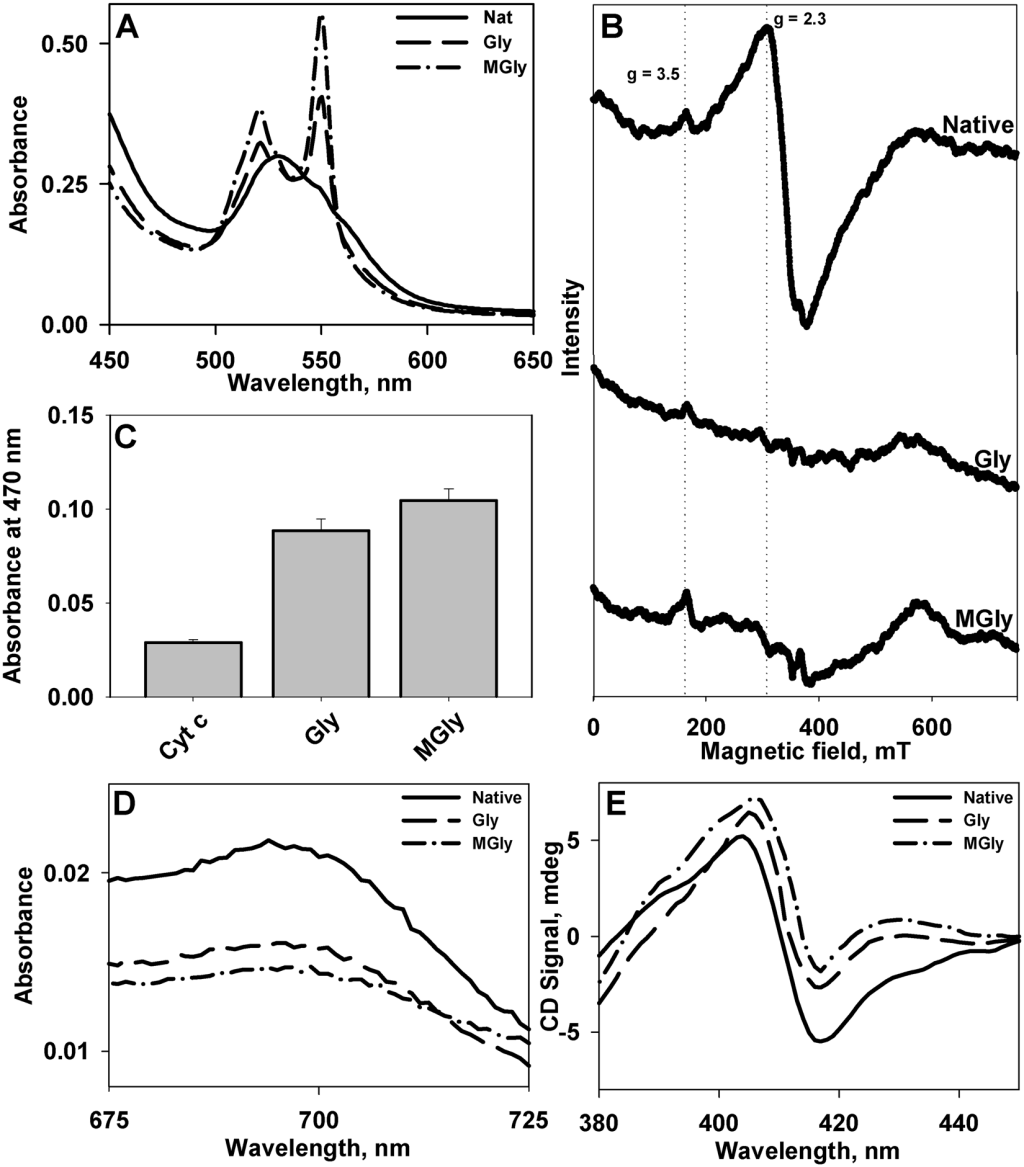
Heme status of Cyt c upon glycation. Visible absorption (**A**) and EPR (**B**) spectra of native and glycated Cyt c. Panel C shows the peroxidase activity of native and glycated Cyt c. Panel D, E show the absorption and soret CD spectra of native and modified Cyt c. Only the spectra of unmodified and Cyt c modified with 10 mM glyoxals are shown.

Exposure of heme upon glycation could in turn affect the redox state of Cyt c. To verify this, we assessed the redox status of glycated Cyt c. We found that the heme centre has been reduced upon modification. Visible absorption spectra of reduced form of Cyt c is defined by two distinct bands at 520 and 550 nm, while oxidized Cyt c exhibits a single broad band with maximum at 530 nm, as also observed in the present study (Fig. 3A). Furthermore, electron paramagnetic resonance (EPR) analyses confirmed reduction of the heme moiety (Fig. 3B). The EPR spectrum of native Cyt c in its oxidized state consists of two signals, g = 2.3 and g = 3.5^18,19^, which were intact for unmodified protein. Upon glycation, these bands disappeared, suggesting the conversion of oxidized Cyt c to reduced form. Furthermore, it was also found that glycation led to activation of peroxidase-like function of Cyt c (Fig. 3C and Fig. S1), which is a crucial step for Cyt c release during intrinsic apoptotic pathway.

### Cyt c Heme-Met80 ligation upon glycation

Alterations in heme iron sixth ligand (Met80) are known to bring about certain affects, both in structural and functional properties of Cyt c. To evaluate the heme-Met80 interaction, we carried out visible absorption and soret CD measurements. Figure 3D, E shows the visible absorption and soret CD spectra of unmodified and modified Cyt c. It is seen that there is disruption of the 695 nm (visible absorption) and 416 nm (soret CD) bands in case of modified Cyt c suggesting loss of heme-Met80 interactions in modified proteins. Under normal conditions, heme in Cyt c exists in hexa-coordinated state. The 695 nm absorbance band is assigned diagnostic characteristic for Met80 coordination to heme moiety. Alteration (or disruption) of this axial ligation lead to loss of this band^20^. Indeed heme-Met80 interaction is disrupted upon glycation and Cyt c undergoes conformational transition as evidenced by decrease in absorption band at 695 nm. This alteration was further probed using another spectroscopic signature, soret CD measurement. The negative dichroic band at 416 nm has also been designated to heme-Met80 ligation^21^ which is lost in presence of glycating agents. Our results indicate that the non-native structure induced by glycation is a penta-coordinate structure with disrupted tertiary interactions.

### Modified Cyt c exhibits AGEs-specific fluorescence characteristics upon prolonged incubation with appearance of non-amyloidogenic structures

**Figure 4:**
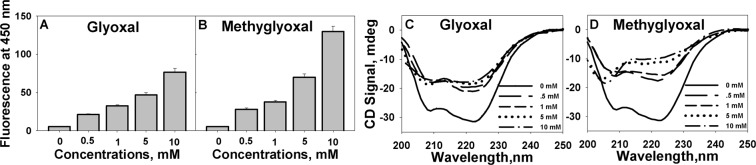
AGEs fluorescence and conformational status of glycated Cyt c upon prolonged incubation. AGEs fluorescence of Cyt c upon treatment with Gly (**A**) and MGly (**B**). Far-UV CD spectra of Cyt c procured after two weeks incubation with Gly (**C**) and MGly (**D**).

**Figure 5:**
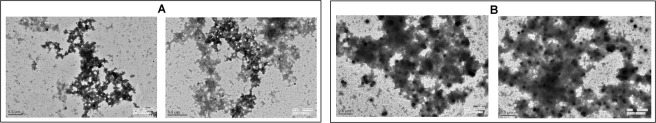
Transmission electron micrographs Cyt c. TEM images of glycated Cyt c incubated for two weeks with 10 mM Gly (**A**) and MGly (**B**).

Fluorescence measurements after incubation for two weeks revealed that modified Cyt c exhibited AGE-specific fluorescence. Formation of AGEs was monitored using excitation and emission at 350 nm and 450 nm respectively^22^. Increase in fluorescence intensity at 450 nm points towards the formation of AGEs upon glycation (Fig. 4A, B). The conformational alterations upon prolonged incubation were further assessed using far-UV CD measurements (Fig. 4C, D). It is seen that the negative peaks at 208 nm and 222 nm, which are the characteristic features for α-helix, is gradually lost with increasing concentration of glyoxals. Secondary structure estimation upon extended incubation of Cyt c with Gly and MGly, suggests a prominent transition of the α-helical components to more unordered structures with increased β-sheet structures (in case of MGly) (Fig. S2). The morphological features of modified proteins were further assessed using TEM. TEM images (Fig. 5) revealed that upon 2-week incubation, Gly-modified Cyt c exists as clusters of spherical structure, whereas MGly-modified Cyt c exists as amorphous structure. However, no amyloidogenic transitions were observed.

## Discussion

Glycation preferably targets the side chains of proteins, particularly Lys and therefore Lys-rich proteins could be the major targets for glycation. Thus, Cyt c (with 19 Lys residues) could be a prime target for such modifications. Our results on conformational assessments suggest that upon glycation, Cyt c attains a non-native state characterized by perturbed tertiary interactions and partially exposed heme. Furthermore, heme in the resultant non-native Cyt c was found to be in a penta-coordinated state with disrupted heme-Met80 ligation. Such structural transition may have important physiological consequences. Since Cyt c is a protein essential for intrinsic apoptotic pathway, and the conformational alterations in this protein has been shown to be responsible for intrinsic apoptotic activity, it is important to examine if the non-native intermediate is an apoptotically-competent species. Cardiolipin (CL) oxidation due to the activation of Cyt c peroxidase function is believed to be primary event towards early intrinsic apoptotic pathway leading to release of Cyt c from IMM. We measured the peroxidase activity of modified Cyt c using two different substrates. It was observed that modified Cyt c exhibits peroxidase activity, but not the native protein. Similarly, ribose-5-phosphate (R5P) has also been shown to induce certain alterations in Cyt c. However, peroxidase activation required prolonged incubation period (one week or more)^23^. In addition, modification of Cyt c by homocysteine thiolactone, accumulated under hyperhomocysteinemia has also been shown to induce gross conformational alteration leading to reduction of heme moiety and peroxidase activation^24^. We conclude that these early structural intermediate represents an apoptotically-competent conformation and therefore hint towards possible involvement of such post-translationally modified Cyt c in apoptosis. In support, many studies using different cell lines showed that Gly and MGly induce apoptosis when provided exogenously^25–29^. Furthermore, concentrations of both compounds in mitochondria are elevated under hyperglycemic conditions and contribute to mitochondrial dysfunction associated with diabetes and aging^30^. In addition to this, it has been demonstrated that disruption of Cyt c heme coordination is responsible for mitochondrial injury during ischemia^31^. Taken together, these results therefore indicate that modification of Cyt c by glycation might be associated with mitochondrial injury leading to apoptosis.

Since glycation is known to result in generation of free radicals^32^, redox state of Cyt c heme could also be altered upon such modifications. To examine the redox status, we performed visible absorption and EPR spectral measurements of the native and modified proteins. Our absorption and EPR spectral measurements revealed that the heme centre of modified Cyt c was rendered reduced upon glycation. The electrostatic interaction between Cyt c and CL is believed to be responsible for anchoring the protein to IMM and optimizes ET between complex III and complex IV^31^. In fact, glycation by R5P has also been shown to weaken the ability of Cyt c to transfer electrons in respiratory pathway and to bind membranes^23^. Hence, it is possible that modifications that lead to reduction of heme in Cyt c would thereby not only disrupt normal functionality of the protein in ET process and cellular energy demands, but also help the protein shed off from IMM.

A number of studies have shown the involvement of different proteins in generation of AGEs and their relation with protein aggregation^9,22,33^. In addition, the presence of high levels of AGEs in brains of patients with neurorogical conditions and their association with amyloid deposition have also been reported^34,35^. In fact, glycation has been shown to enhance the severity linked with neurotoxicity of Aβ_1–42_ peptide^36^. Extended incubation (two weeks) of Cyt c with Gly and MGly resulted in rapid increase in AGEs fluorescence (see Fig. 4A, B). Since AGEs formation has been associated with various clinical complications and human pathological conditions, including NDs^37–41^, it is very likely that AGEs formation induced by these agents could also contribute to these pathologies. We have further analysed the morphological nature of glycated Cyt c. Our TEM studies of Gly-modified Cyt c suggest generation of spherical structures with varying sizes, with diameters ranging from 50–100 nm (Fig. 5A). These structures do not have any specificity towards amyloids specific dye (ThT) (Fig. S3), suggesting that these species are non-amyloidogenic. However, MGly-modified Cyt c showed disordered amorphous structures (Fig. 5B). The variation in nature of aggregates could be due to the difference in structural alterations induced by these two molecules. Far-UV CD measurements (Fig. 4D) revealed that MGly induced significant alterations in secondary structural element with appearance of prominent disordered-like character. The 208 nm and 222 nm bands, characteristic signature of α-helical structure were completely diminished in presence of MGly. At higher concentrations of MGly used (5 and 10 mM), there was the appearance of new signal around 204 nm suggesting that the modified protein now exists as an unordered state. In case of Gly, there were disruptions of α-helical structures, but no appearance of disordered state. Thus, differences on the effect of the modifying agents on secondary structural levels could be one reason for the differences in final state of aggregates. Similarly, glycation (by D-ribose) has been shown to generate globular aggregates in case of α-synuclein^42^. No fibrillar or amyloidogenic structures were observed. In case of insulin, glycation by MGly was shown to reduce insulin fibrillation, leading to formation of “native-like” aggregates^12^. It is suggested that modification led to a less compact and less stable structure which may be associated to increased dynamics, preventing the formation of rigid cross-β core structure found in amyloid fibrils. The study pointed that MGly could trigger a drifting from an amyloidogenesis towards a “native-like” aggregation pathway, a mechanism that might be important in the context of the amyloidogenicity of AGE-modified proteins involved in conformational diseases^12^.

Our study demonstrates the role of glycation-induced early structural alterations and gain of peroxidase function in Cyt c can lead to CL oxidation, hence provides a hint for Cyt c release. Furthermore, prolonged incubation of Cyt c with glyoxals generated AGEs which are also associated with several neurological disorders. Analyses of these AGEs via TEM confirmed that these species exist as clustered spherical or amorphous structures. Studies on how conformational alterations in the protein induce mitochondrial injury could yield useful insights in understanding the mechanism of early apoptotic pathway.

## Experimental Procedure

### Materials

Cyt c (from bovine heart), and other chemicals were purchased from Sigma-Aldrich. Protein solution was dialyzed extensively against 0.1 M KCl at pH 7.0 at 4 °C. Cyt c was oxidized using 0.01% potassium ferrocyanide before dialysis. All solutions for optical measurements were prepared in degassed buffer (0.05 M phosphate buffer, pH 7.4).

### Protein modification

For protein modification, Cyt c was incubated in presence of varying concentrations of Gly and MGly (0–10 mM) in 0.05 M potassium phosphate buffer, pH 7.4 at 37 °C. These treated/untreated protein samples were further used for subsequent analyses.

### Carbonyl Estimation

Carbonyl content in control and modified proteins were assayed by method described by Levine *et al*.^43^. Briefly, an aliquot of treated Cyt c were incubated for 1 hr at room temperature with DNPH (0.1% w/v in 2 N HCL). The reaction was stop by addition of equal volumes of 20% trichloroacetic acid (TCA) and centrifuged to obtain a pellet. DNPH was removed by extracting the pellets two times using 1 ml of ethyl acetate:ethanol (1:1 v/v) solution. Pellets were dried and dissolved in 6.0 M GdmCl (pH 7.0). Solubilized hydrazones were measured at 370 nm and concentration of DNPH derivatized proteins was determined using molar extinction coefficient of 22,000 M^−1^ cm^−1^.

### Intact Mass analysis

The treated samples were analyzed using an Applied Biosystems/MDS Sciex 4800 Plus MALDI TOF/TOF Analyzer to obtain the MALDI spectra. Modified protein solutions were mixed with sinapic acid matrix in 1:1 ratio and kept to dry. The protein concentration used was 80 µM.

### CD Measurements

CD spectra were procured in Jasco J-810 spectropolarimeter equipped with Peltier-type temperature controller with three accumulations. Protein concentration used for CD measurements were 15–20 µM. Cells of 1 and 10 mm path lengths were used for measurements of far- and near-UV CD spectra, respectively. For Soret CD measurements, cell of 1.0 cm path lengths was used. Necessary blanks were subtracted for each measurement.

### UV-Visible Spectrophotometry

Absorption spectra of were recorded in a Jasco V-660 spectrophotometer equipped with Peltier-type temperature controller. The protein concentration used for spectral measurements was 15 µM. For the 695 nm absorption band, protein concentration used was 50 µM. For all measurements, cell of 1.0 cm path length was used.

### Electron Paramagnetic Resonance (EPR)

EPR measurements were carried out in a Bruker EMX MicroX spectrometer. Following conditions were maintained for the measurements: gain, 1 × 10^3^; modulation amplitude, 4.0 G; microwave power, 16 mW; temperature, 298 K; conversion time, 20 ms; and time constant, 655 ms. Protein samples were loaded in sealed quartz capillary tubes and transferred to EPR cavity to procure spectra. The protein concentration was kept at 150 µM.

### Fluorescence Measurements

Fluorescence spectra were measured in a Perkin Elmer LS 55 Spectrofluorimeter in a 3 mm quartz cell, with both excitation and emission slits set at 10 nm. Protein concentration for all experiments was 5 µM. For AGEs fluorescence measurements, excitation and emission wavelengths were 300 and 450 nm, respectively. ANS was excited at 360 nm and emission collected at 400–600 nm range and ANS concentration was kept 75 µM. For ThT binding assay, samples were excited at 450 nm and emission collected at 475–600 nm range. ThT concentration was 25 µM. Necessary blanks were subtracted for each sample.

### Peroxidase activity assay

Peroxidase activity was assayed with guaiacol by measuring absorption at 470 nm for tetraguaiacol formed as product^44^. Protein concentration was kept 1 μM, 1 mM guaiacol, and 2 mM H_2_O_2_. The reactions were also repeated with ABTS and H_2_O_2_ (100 µM and 1 mM respectively) by measuring absorption at 415 nm^45^.

### TEM imaging

Modified Cyt c solutions were placed on copper grid and left to dry at room temperature for 5 min. Negative staining was performed using 1.0% uranyl acetate and allowed to air dry. The samples were examined using FEI Tecnai G2-200kV HRTA TEM (Netherland) operating at 200 kV.

## Supplementary information

Supplementary Information
