# Supplementary material for: Covalent Modification by Glyoxals Converts Cytochrome c Into its Apoptotically Competent State
Source: Sci Rep. 2019 Mar 18;9:4781. doi: 10.1038/s41598-019-41282-2 (PMC6423144; doi:10.1038/s41598-019-41282-2)
Supplement: Supplementary file 1 — Supplementary Information [file 41598_2019_41282_MOESM1_ESM.pdf]

SUPPLEMENTARY INFORMATION

Covalent Modification by Glyoxals Converts Cytochrome c Into its Apoptotically Competent State

Gurumayum Suraj Sharma[1,2], Marina Warepam[3], Reshmee Bhattacharya[1] and Laishram Rajendrakumar Singh[1]*

[1]Dr. B. R. Ambedkar Center for Biomedical Research, University of Delhi, Delhi-110007, India

[2]Department of Botany, Zakir Husain Delhi College, University of Delhi, New Delhi-110002, India

[3]Department of Biotechnology, Manipur University, Canchipur, Imphal East, Manipur-795003

***Correspondence Author:** lrsingh@acbr.du.ac.in, lairksingh@gmail.com (Dr. Laishram Rajendrakumar Singh)

Mobile: +91 9811630757

Running Title: Structural and Functional Status of Cytochrome c Upon Glycation.

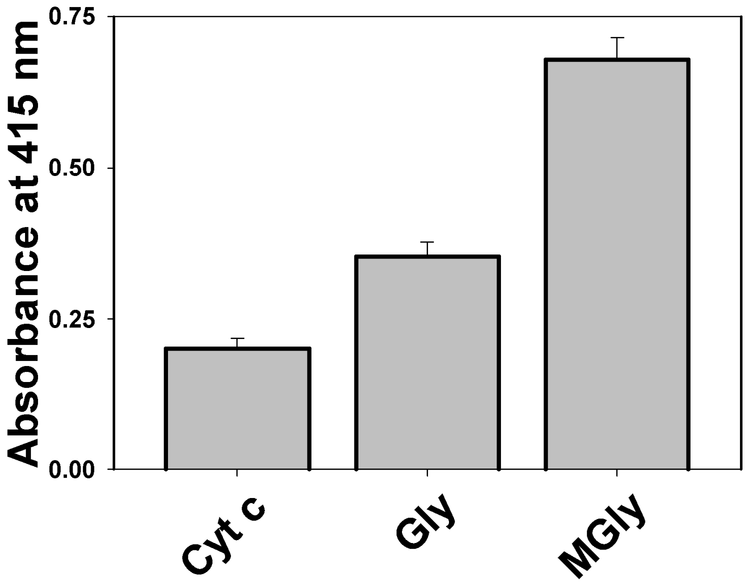

Figure S1: Peroxidase activity of native and modified Cyt c. Activity was measured with ABTS and H_2O_2 (100 µM and 1 mM respectively) by flowing the absorption at 415 nm.

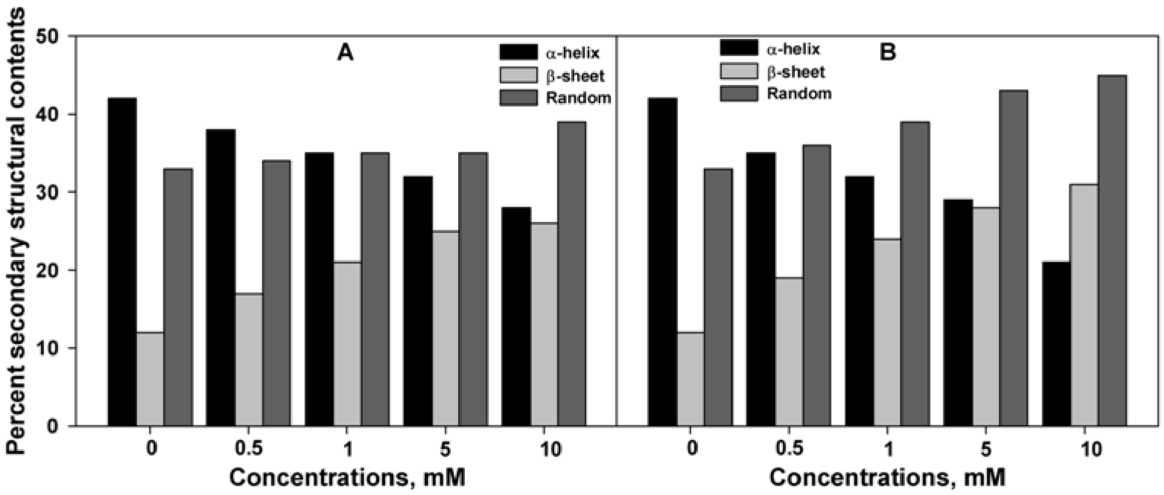

Figure S2: Secondary structural composition of Cyt c modified with increasing concentration of Gly and MGly (0-10 mM).

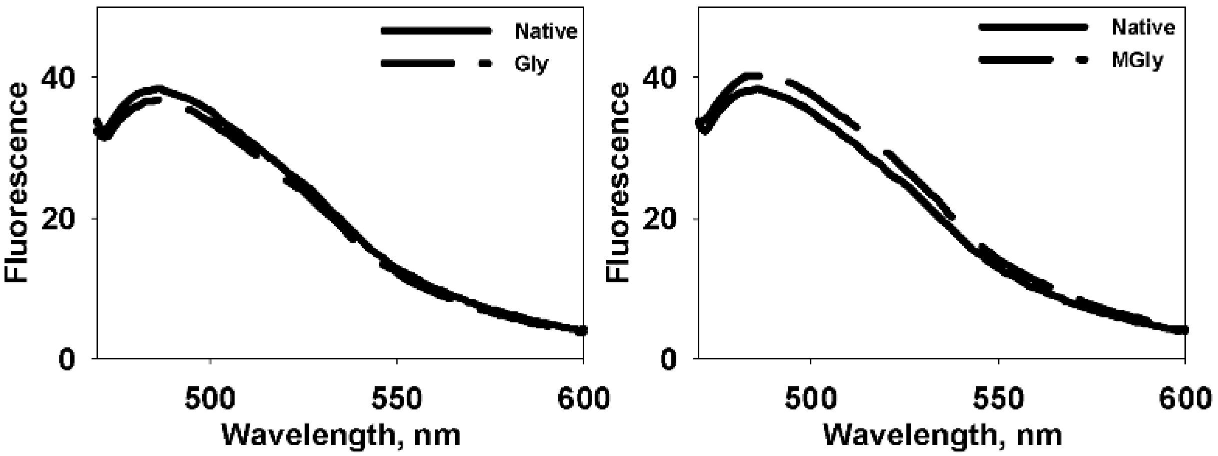

Figure S3: ThT fluorescence spectra of native and modified Cyt c. Only spectra of native and Cyt c modified with 10 mM glyoxals are shown.
